# Past and Present of Point-of-Care Ultrasound (PoCUS): A Narrative Review

**DOI:** 10.7759/cureus.50155

**Published:** 2023-12-08

**Authors:** Nikhila Chelikam, Ankit Vyas, Rutikbhai Desai, Nida Khan, Karanrajsinh Raol, Anusha Kavarthapu, Prahasith Kamani, Garad Ibrahim, Sowmya Madireddy, Suveenkrishna Pothuru, Parth Shah, Urvish K Patel

**Affiliations:** 1 Clinical Research, Icahn School of Medicine at Mount Sinai, New York, USA; 2 Internal Medicine, Baptist Hospitals of Southeast Texas, Beaumont, USA; 3 Community Medicine, Gujarat Medical Education and Research Society (GMERS) Medical College and Hospital, Ahmedabad, IND; 4 Internal Medicine, Jinnah Sindh Medical University, Karachi, PAK; 5 Internal Medicine, St. Vincent's Medical Center, Bridgeport, USA; 6 Internal Medicine, Gujarat Medical Education and Research Society (GMERS) Medical College and General Hospital, Gandhinagar, IND; 7 Internal Medicine, Richmond University Medical Center, Staten Island, USA; 8 Internal Medicine, Gandhi Hospital, Hyderabad, IND; 9 Internal Medicine, Hennepin County Medical Center, Minneapolis, USA; 10 Internal Medicine, Mamata Medical College, Khammam, IND; 11 Internal Medicine, Ascension Via Christi Hospital, Manhattan, USA; 12 Hospital Medicine, Tower Health Medical Group, Reading, USA; 13 Public Health and Neurology, Icahn School of Medicine at Mount Sinai, New York, USA

**Keywords:** pocus in inpatient setting, pocus in outpatient setting, ultrasound types, ultrasound guided imaging, covid-19, pocus (point of care ultrasound)

## Abstract

This article aims to conduct a literature review to gain insight into point-of-care ultrasound (PoCUS). PoCUS is a rapid, accurate, non-invasive, and radiation-free imaging modality that can be used in stable and unstable patients. PoCUS can be performed parallel to physical examination, resuscitation, and stabilization; repeated exams in critical patients are essential for improving sensitivity. The review highlights how PoCUS, which was initially used to detect free intraperitoneal fluid in trauma patients, has developed into a life-saving diagnostic tool that could be utilized by treating physicians during various stages of diagnosis, resuscitation, operation, and postoperative critical care when managing sick patients. The review also notes the barriers to the widespread uptake of PoCUS in general internal medicine and the recent commercial availability of "pocket" or handheld probes that have made PoCUS more readily available. This review concludes that adopting a focused binary decision-making approach can maximize PoCUS's value in many clinical settings, including emergency departments, intensive care units, and operation theatres. Overall, the review emphasizes the importance of awareness of common indications, limitations, and strengths of this evolving and promising technology to determine its future trajectory: Providing comprehensive PoCUS training within internal medicine curriculums and supporting trainers to do so.

## Introduction and background

Over the last decade, ultrasound imaging and information systems have become sophisticated and digital, improving accessibility and affordability for ultrasound examinations. The ultrasound equipment for emergency care has become more versatile, portable, reduced in size, and predominant for better patient outcomes. This evolution allows us to do instant reporting via wireless connectivity over the electronic medical records (EMR) and picture archiving and communication system. These new devices are currently being evaluated in various clinical settings and more diverse situations that were not previously possible [1].

Point-of-care ultrasound (PoCUS) is a rapid, repeatable, accurate, inexpensive, non-invasive, and radiation-free imaging technique used in stable and unstable patients. It may also be performed parallel to physical examination, resuscitation, and stabilization. Performing repeated ultrasound exams in critical patients is essential and improves its overall sensitivity [2]. The use of PoCUS by non-radiologists has developed over time. Initially, it was used to detect free intraperitoneal fluid in multiple trauma patients and was termed focused assessment sonography of trauma [3]. In non-radiologists's hands, PoCUS developed into a life-saving diagnostic tool physicians use at all levels of patient care, including diagnosis, resuscitation, operation, and postoperative critical care. Today, there is enough evidence to show that PoCUS is an effective diagnosis tool, even for non-radiologists [3]. The consensus-based recommendations by the Canadian Internal Medicine Ultrasound group provided a framework for training programs at a national level with four and seven PoCUS applications and three and four ultrasound-guided procedures for PGY 1-3 and PGY 4-5, respectively [4].

PoCUS is a study that allows for the evaluation of shock status and is also an on-the-spot clinical decision tool that facilitates critical decision-making in emergencies in a short amount of time. PoCUS is unique with expanding indications to study different organs in a systematic approach at the same time and thus can be an extension of the clinical examination. For example, a simple, dichotomous protocol that uses a single microconvex probe without the need for advanced techniques helps diagnose acute respiratory failure [5]. PoCUS is safe and repeatable, and when used by trained acute care professionals, it functions like a stethoscope. As mentioned above, these characteristics of PoCUS make it completely different from routine radiological studies [3]. These advantages make PoCUS valuable in many clinical settings, including emergency departments, intensive care units (ICU), and operation theatres. With increased interest, training, and experience, PoCUS will be more prominent in diagnosing medical conditions [3]. Significant barriers to the widespread uptake of PoCUS within general internal medicine remain for several reasons. Part of this is likely a lack of understanding of the evidence bases for this imaging modality. It is also likely that clinicians place undue confidence in the traditional clinical examination, which evidence suggests is often less robust than thought [6].

"Pocket, or handheld probes," currently commercially available, have made PoCUS less expensive and more accessible. Their web-based operation and internet connectivity could facilitate the introduction of on-the-job competency assessment and remote learning programs. Remote teaching and image review capabilities also promote quality assurance and reassurance without direct supervision when bridging the gap between initial PoCUS training and the development and monitoring of proficiency. These pocket probes offer low initial costs, low maintenance, and remote viewing of acquired images via a cloud-based platform. The availability of machines, portability, cost, remote viewing, and telemedicine are no longer barriers to PoCUS adoption. Through artificial intelligence (AI) and augmented reality, both novices and experts can acquire and interpret images, leading to faster diagnosis, focused clinical care, and better clinical decision-making. A recent study mentioned the importance of utilizing PoCUS in the primary healthcare system and PoCUS's positive impact on cost-effectiveness [7]. These handheld ultrasound devices will be essential to implementing PoCUS in daily practice, which is no longer a possibility but a certainty. Handheld ultrasound probes are valuable because of their portability, cloud-sharing, and telemedicine capabilities. These features could improve patient care in the peri-operative period and faculty and resident PoCUS education. Physicians benefit from a pocket-sized probe that can switch from curved to linear image acquisition and low- to high-frequency imaging [8]. PoCUS may yield helpful information during damage control resuscitation on the operating table after the damage control laparotomy and before transferring the patient to the ICU [9].

Experience over the last 25 years has shown that PoCUS is a handy tool when used by non-radiologists. Understanding PoCUS's limitations and adopting a focused binary decision-making approach can maximize its value to answer specific questions without going into detailed radiological studies. PoCUS has become an extension of the clinical examination [2]. Evidence supports that adding PoCUS to examining selected patients leads to improved and earlier diagnosis in a hospital setting [10]. While many studies have shown a positive impact of PoCUS on promoting medical care and reducing morbidity, mortality, and overall healthcare costs, its uniform implementation appears to be limited across the US healthcare system. Limitations can be attributed to various barriers, such as lack of training, resource scarcity, and low reimbursement.

Training primary care physicians and emergency care providers in general is the key to improving PoCUS use [11]. With the aid of portable ultrasound devices, PoCUS has widened its scope to many subspecialties beyond critical care and emergency medicine. We reviewed the literature to understand its use in appropriate settings, subspecialties, and the COVID-19 pandemic. Awareness of the evolving and promising technology's common indications, limitations, and strengths is essential to determine the future trajectory.

## Review

Traditional use of ultrasound

**Figure 1:**
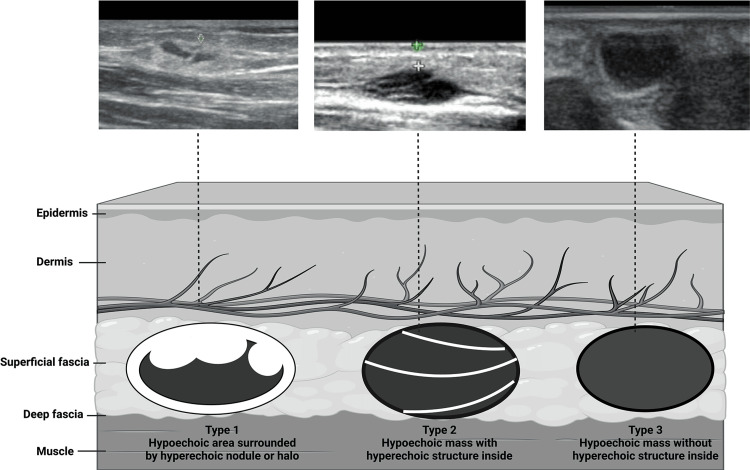
Anechoic: Structures appear black, meaning no internal echoes. Examples: cysts, vessels, gallbladder ascites, and water. Hypoechoic: Gives off fewer echoes; they are darker than surrounding structures. Example: lymph nodes and tumors. Hyperechoic: Increased density of sound waves compared to surrounding structures. Example: bone and fat calcifications Credit: https://onlinelibrary.wiley.com/doi/10.1002/jcu.23542

Ultrasound is a non-invasive tool used in many ways for decades in the medical field. Diagnostic ultrasound can non-invasively produce images of internal organs inside the body, although it is ineffective in visualizing bones or tissues containing air like the lungs. However, in certain situations, it can produce images of bones (such as in fetuses or small babies) or the lungs and their lining when filled with fluid. One of its most common applications is monitoring fetal growth and development during pregnancy. Additionally, ultrasound imaging is utilized to visualize other areas such as the heart, blood vessels, eyes, thyroid, brain, breast, abdominal organs, skin, and muscles. The resulting ultrasound images can be displayed in 2D, 3D, or 4D (3D in motion) [7]. A tissue's echogenicity refers to its ability to reflect or transmit US waves when surrounded by surrounding tissues. In terms of echogenicity, a structure can be classified into hyperechoic, hypoechoic, or anechoic (black on the screen) (Figure 1). A visible contrast difference will be apparent when structures with different echogenicities are in contact [12].

Karl Dussik (1908-1968), an Austrian neurologist, was the first physician to use ultrasound for medical imaging who attempted to depict changes in brain ventricle size secondary to tumor growth. In an early application of ultrasound technology, transducers were placed on both sides of a patient's partly submerged head [13]. Ultrasound technology has many applications that benefit patients, such as fetal monitoring, joint injections, arterial line placements, and diagnosing bone joint pathology. These uses are popular due to their low cost, portability, and the fact that they do not involve radiation exposure. Moreover, advances in technology and engineering have ended the use of ultrasound beyond imaging and diagnostics, making it a viable therapeutic modality [14].

Types of devices and functionality

Traditional ultrasound devices are available in various types, including portable, compact, and console-based models. Lightweight, maneuverable, portable, and compact devices are designed for point-of-care use. At the same time, console-based models offer advanced imaging capabilities and diagnostic accuracy and are used in various clinical settings. Table 1 mentions different types of devices and their functionality. Table 2 gives an insight into ultrasound utilization in the medical field.

**Table 1:** Types of devices and functionality POC: Point of care

Type of ultrasound model	Description	Advantages	Disadvantages
Portable ultrasound	- Small and lightweight; designed for easy transport and use in various clinical settings, including hospitals, clinics, and emergency medical service settings	- Small size and portability make it ideal for POC use, such as at a patient's bedside or in remote locations where access to traditional imaging equipment may be limited - Useful in emergencies as they can be quickly transported to the scene of an accident or emergency	- Limited image quality due to smaller size and simplified interface, which can make it difficult to diagnose certain conditions accurately - Limited features and capabilities compared to larger console-based systems - Typically, battery dependent, which can limit their use time and require frequent recharging or replacement of batteries
Compact ultrasound	- Smaller and lightweight than console-based models but with more features and capabilities than portable models - Often used in situations where portability is still essential, but advanced imaging features are still needed.	- A wide range of imaging capabilities, such as color Doppler, spectral Doppler, and 3D/4D imaging - Lightweight and easy to transport- Easy to use with intuitive interfaces and user-friendly controls - Designed with the patient’s comfort in mind, with smaller probes and less invasive procedures - Cost-effective compared to console-based ultrasounds, which makes it more accessible to smaller clinics and practices that may not have a budget for larger systems	- Limited imaging capabilities compared to larger console-based devices - Limited ability to perform more advanced procedures, such as biopsies or interventions - Typically, battery dependent, which can limit their use time and require frequent recharging or replacement of batteries
Console-based ultrasound	- Larger and more advanced systems used in hospitals, clinics, and medical centers - Designed to provide high-quality images and diagnostic accuracy for a wide range of medical applications, including cardiology, radiology, obstetrics, and more	- With more advanced technology, they are more accurate in diagnosing conditions than portable or compact models - Ability to perform more advanced procedures such as biopsies or interventions - Better ergonomics - with larger screens, better user interfaces, and more comfortable control panels	- Large size and heavier, which makes it difficult to transport and move around - More expensive than compact or portable models, which may make them less accessible to some healthcare providers and facilities - Longer learning curve due to more advanced technology to use effectively - Typically require a reliable power source, which can limit their use in remote or resource-limited settings

**Table 2:** Utilization of ultrasound in the medical field EP: Emergency physician; ED: Emergency department; FAC: Femoral artery catheterization; MVP: Mitral valve prolapse; OR: Odds ratio; RR: Relative risk; CI: Confidence interval; SN: Sensitivity; SP: Specificity; PPV: Positive predictive value; NPV: Negative predictive value; US: Ultrasonography; ALT: Anatomical landmark-guided technique; FV: Femoral vein; PSV: Peak systolic velocity; ICA: Internal carotid arteries; CCA: Common carotid arteries; EDV: End-diastolic velocity; SROC: Summary receiver operating characteristic; DOR: Diagnostic odds ratio

Study name, year	Study type	Use	Details/outcome	Conclusion
Costantino et al., 2005 [15]	Prospective	- To compare ultrasonographic with traditional approaches using palpation and landmark guidance	- 60 patients - Success rate was greater for the ultrasonographic group (97%) versus the control (33%)	-Ultrasonographic-guided peripheral intravenous access is more successful than traditional “blind” techniques
Mallory et al., 1990 [16]	Prospective	- To compare conventional versus ultrasound-guided vein cannulation techniques	- All consecutive patients who required urgent or urgent-elective internal jugular vein cannulation during the study period - 2D ultrasound was significantly better than conventional guidance in reducing the number of failed site cannulations from 6/17 (35%) to 0/12 (0%), (p < 0.05)	- Intensivists can increase successful internal jugular vein cannulation using ultrasound guidance - 2D ultrasound should be considered for patients with difficulty cannulating or those at high risk of cannulation complications
Hansen et al., 2014 [17]	Observational	- To compare the traditional palpation technique and dynamic needle tip positioning technique	- The first attempt success rate was significantly higher in the ultrasonography dynamic needle tip positioning group (23/40 vs. 38/40, p < 0.001)	- Ultrasonography guidance using dynamic needle tip positioning technique for radial artery catheterization significantly improves clinically relevant aspects of the procedure
Schlager et al., 1994 [18]	Prospective	- To examine the use of limited, goal-directed, 2D ultrasound studies performed by EP; to assess the frequency, variety, and accuracy of their readings	-104-bed community hospital with an ED volume of 25,000 patients annually - Three studies most commonly performed were for gallbladder disease (53%), intrauterine pregnancy (28%), and abdominal aortic aneurysms (7%)	- With appropriate training, EP can perform diagnostic ultrasound studies with a high degree of accuracy
Sobolev et al., 2015 [19]	Systematic review and meta-analysis of RCTs	- To determine the utility of real-time 2D ultrasound guidance for FAC	- 1422 subjects (4 trials); with 703 subjects in palpation group and 719 subjects in ultrasound-guided group - Compared with traditional methods, ultrasound guidance for FAC was associated with 49% reduction in overall complications, including hematoma and accidental venipuncture (RR, 0.51; 95% CI, 0.28-0.91) - Also associated with 42% improvement in likelihood of first-attempt success (RR, 1.42; 95% CI, 1.01-2.00)	- Use of real-time 2D ultrasound guidance for FAC decreases life-threatening vascular complications; improves first-pass success rate
Hershman et al., 1989 [20]	Prospective	- To investigate the use and value of echocardiography in patients suspected of having MVP	- A total of 106 echocardiograms were ordered by 45 different physicians - >80% of all echocardiograms were ordered to address diagnostic or therapeutic concerns - On echocardiography, 47 (44%) patients were found to have MVP, six (6%) had mitral regurgitation without prolapse, and 53 (50%) had normal results - Echocardiographic results led to a change in diagnosis in 59 (56%) patients - Change in management occurred in 29 (27%) patients, with 25 of these 29 changes (86%) related to the initiation or discontinuation of antibiotics	-Echocardiography frequently alters diagnostic assessments and leads to therapeutic changes in some patients suspected of having MVP
Dietz et al., 2001 [21]	Prospective	- To evaluate the use of ultrasound in the quantification of prolapse and compare findings with clinical assessments	- Clinical staging and International Continence Society coordinates were obtained for all 145 patients, as were ultrasound coordinates for descent of anterior and posterior vaginal walls - 18% of uteri of those women who had not had a hysterectomy in past could not be seen; none of these women suffered from uterine prolapse clinically - Correlation with prolapse assessment system recently endorsed by International Continence Society was good (r = 0.77 for uterine prolapse, r = 0.72 for anterior vaginal wall and r = 0.53 for posterior vaginal wall descent)	- Trans labial ultrasound can be used to quantify female pelvic organ prolapse
Healey et al., 1996 [22]	Prospective	- To evaluate the utility and feasibility of abdominal ultrasound in blunt trauma patients	- 800 ultrasound studies were performed over 15 months. - The average time to arrival of ultrasound was 17.3 minutes (range 0-120), and the average minutes to start after arrival was 7.0 (range 1-49) - The average time required to perform the study was 10.6 minutes (range 2-26)	- Ultrasound can be obtained rapidly, integrated into the resuscitation, and completed quickly
Smith-Bindman et al., 2013 [23]	Retrospective	- To quantify the risk of thyroid cancer associated with thyroid nodules based on ultrasound	- 8806 patients underwent 11618 thyroid ultrasound examinations during the study period, including 105 patients diagnosed as having thyroid cancer (incidence of 0.9 cancers per 100 ultrasound examinations - Cancers were diagnosed one day to 6.1 years after ultrasound imaging, and among control patients, there was a mean follow-up of 4.2 years (range, 2.0-10.9 years)	- Thyroid ultrasound imaging could be used to identify patients who have a low risk of cancer for whom biopsy could be deferred
Manning et al., 1995 [24]	Prospective	- To determine the ability of transesophageal echocardiography to identify or exclude left atrial thrombi accurately	- 231 consecutive patients having transesophageal echocardiography before elective repair or replacement of mitral valve or excision of a left atrial tumor - 56% of patients had a history of atrial fibrillation; 17% had a history of thromboembolism	- Transesophageal echocardiography is highly accurate for identifying left atrial thrombi and can be used clinically to exclude left atrial thrombi
Rahimzadeh et al., 2022 [25]	Clinical trial	- To compare the effect of ultrasonography, a traditional method, on the success rate of spinal anesthesia	- Success rate of dural puncture at the first attempt of entry and time required to determine needle entry site in ultrasonography group (55.2%) was significantly higher than that in Landmark group (21.4%) (p < 0.05) - Time required for needle entrance to CSF exit, total procedure time for patients, number of needles redirection without complete removal of skin, and number of needle entry after complete removal of the skin in ultrasonography group was significantly lower than that in Landmark group (p < 0.05)	- Use of ultrasonography in comparison with the traditional method has been effective on the success rate of spinal anesthesia by an anesthesia resident
Berghella et al., 1997 [26]	Prospective	- To compare the accuracy of ultrasonographic and manual cervical examinations for the prediction of preterm delivery	- Excluding six induced preterm deliveries, 96 pregnancies were analyzed - Mean cervical length measured by ultrasonography was 20.6 mm in pregnancies delivered preterm (n = 17); 31.3 mm in pregnancies delivered at term (n = 79) (p = 0.003); mean cervical lengths measured by manual examination were 16.1 mm and 18.6 mm in same preterm and term pregnancies, respectively (not significant) - 16th and 25th-week ultrasonographic cervical lengths predicted preterm delivery most accurately (p < 0.0005) - 25th percentiles of ultrasonographic (25 mm) and manual (16 mm) cervical lengths showed relative risks for preterm delivery of 4.8 (95% CI l 2.1 to 11.1, p = 0.0004) and 2.0 (95% CI 0.5 to 4.7, p = 0.1), respectively; SN, SP, and PPV and NPVs were 59%, 85%, 45%, 91%, and 41%, 77%, 28%, and 86%, respectively	- Cervical length measured by ultrasonography is a better predictor of preterm delivery than is cervical length measured by manual examination - Cervical ultrasonography in patients at high risk for preterm birth seems to be most predictive of preterm delivery when it is performed between 14 to 22 weeks gestation
Prabhu et al., 2010 [27]	Clinical trial	- To determine if US-guided insertion was superior and safer than ALT for the FV	- Both groups were comparable regarding age, gender of patients, operator experience, and side of catheterization - Overall success rate was 89.1%, with 80% using ALT and 98.2% under US guidance (p = 0.002) - First attempt success rate was 54.5% in ALT group compared to 85.5% in USG group (p = 0.000) - Complication rate was 18.2% in ALT group; 5.5% in USG group (p = 0.039) - OR for complications with two or more attempts =10.73 with a RR of 3.2. - OR for successful insertion using USG = 13.5 (95% CI: 1.7 to 108.7)	- US guidance significantly improves the success rate, reduces the number of attempts, and decreases the incidence of complications related to FV insertion
Carpente et al., 1995 [28]	Retrospective	- To develop duplex criteria for determination of 60% or greater carotid artery stenosis by comparison with arteriography	- Criteria determined for detection of 60% or greater stenosis were as follows: PSV_ICA_ >170 cm/sec (SN 98%, SP 87%, PPV 88%, NPV 98%, accuracy 92%), EDV_ICA_ >40 cm/sec (SN 97%, SP 52%, PPV 86%, NPV 86%, accuracy 86%), PSV_ICA_/PSV_CCA_ >2.0 (SN 97%, SP 73%, PPV 78%, NPV 96%, accuracy 76%), EDV_ICA_/EDV_CCA_ >2.4 (SN 100%, SP 80%, PPV 88%, NPV 100%, accuracy 88%) - If all of above criteria were met, 100% accuracy was achieved	- Duplex criteria can reliably determine >60% carotid artery stenosis. - Use is appropriate to specific clinical situations of patient screening for lesions (high SN and NPV) or as a sole preoperative imaging modality (high PPV)
Chou et al., 2015 [29]	Systematic review and meta-analysis	- To summarize evidence on the diagnostic value of ultrasonography for assessment of endotracheal tube placement in adult patients	- 12 eligible studies involving adult patients and cadaveric models were identified from 1488 references - For detection of esophageal intubation, pooled SN was 0.93 (95% CI: 0.86-0.96), and the SP was 0.97 (95% CI: 0.95-0.98); area under the summary ROC curve was 0.97 (95% CI: 0.95-0.98) - Positive and negative likelihood ratios were 26.98 (95% CI: 19.32–37.66) and 0.08 (95% CI: 0.04-0.15), respectively	- Current evidence supports that ultrasonography has high diagnostic value for identifying esophageal intubation - With optimal SN and SP, ultrasonography can be a valuable adjunct in this aspect of airway assessment, especially in situations where capnography may be unreliable
Llamas-Álvarez et al., 2017 [30]	Systemic review and meta-analysis	- To assess the accuracy of bedside lung ultrasonography for diagnosing pneumonia in adults	- 16 studies (2,359 participants); there was significant heterogeneity of both SN and SP according to the Q test, without clear evidence of threshold effect -Area under the SROC curve was 0.93, with a DOR at an optimal cut point of 50 (95% CI, 21-120) - A tendency toward a higher area under the SROC curve in high-quality studies was detected; these differences were not significant after applying bivariate meta-regression	- Lung ultrasonography can help accurately diagnose pneumonia, promising as an adjuvant resource to traditional approaches

Utilization spectrum of PoCUS

PoCUS has recently emerged as a technology with a wide array of uses. Being widely used in emergency rooms and diagnosing multiple medical conditions, its scope of utilization has broadened [31]. A prospective clinical trial shows that PoCUS guides fluid resuscitation in critically ill patients [32]. Zanobetti et al. regarded it as a reliable source of diagnosis for dyspnea in patients with cardiac and pulmonary conditions [33]. A retrospective observational study proved that it helped prompt diagnosis and management changes for various conditions, aiding with the targeted medications and intervention use [34]. Daily use of PoCUS in the morning rounds reduced the length of hospital stay, ICU stay, and length of mechanical ventilation in the various patients [35]. The PoCUS role expanded in obstetrics and gynecology by aiding in lung pattern recognition in pregnant patients to cephalic presentations, fetal positions, and free fluid accumulations in busy medical settings [36,37]. Studies by Akyol et al. and Becker et al. prove the high sensitivity of PoCUS in shoulder dislocations and mild sensitivity even in small bowel obstructions, respectively [38,39]. Studies also emphasize its role in detecting ankle fractures, skin and soft tissue infections, and peritonsillar abscesses [40-42]. Aiding in decreasing the role of CT in diagnosing acute appendicitis and effective diagnosis of neck masses in children, PoCUS's scope is widening in the field of internal medicine and various other specialties [43,44]. Table 3 gives an insight into PoCUS utilization in the medical field.

**Table 3:** Current utilization spectrum of PoCUS cIVC: Inferior vena cava collapsibility; LV: Left ventricle; RV: Right ventricle; COPD: Chronic obstructive pulmonary disease; SP: Specificity; SN: Sensitivity; FEEL: Focused echocardiographic evaluation in life support; SBO: Small bowel obstruction; ED: Emergency department; ECA: Enteric-coated aspirin; PEG: Polyethylene glycol; PIS: Pulmonary interstitial syndrome; ONSD: Optic nerve sheath diameter; LVEDP: Left ventricular end-diastolic pressure; BNP: Brain natriuretic peptide; ECG: Electrocardiogram; ECMO: Extracorporeal membrane oxygenation; EP: Emergency physician; LOS: Length of stay; PoCUS: Point-of-care ultrasound

Study name, year	Study type	PoCUS use	Study details/outcome	Conclusion
Cardiovascular Disorders				
Corl et al., 2017 [32]	Prospective	- To determine the ability of cIVC to identify spontaneously breathing critically ill patients’ response to additional intravenous fluids administration	- 124 critically ill patients - cIVC detected responsiveness: area under the curve =0.84 (0.76, 0.91) - Optimum cutoff of 25% rather than 40% in cIVC decreased misclassification	- cIVC helped in differentiating non-fluid responders from fluid responders - Can help guide intravenous fluid resuscitation among critically ill patients
Colclough et al., 2017 [45]	Prospective, single center	- Comparison between PoCUS use (V scan device) vs. control group performed with randomization - Focus echocardiography within 10 minutes and followed by final diagnosis, length of stay, in-patient mortality, and 30-day mortality	- 52 patients of age >18 years presented with shortness of breath - 33% abnormal scan; 9% LV hypertrophy, 9% moderate and 14% severe impaired systolic LV function, 9% abnormal systolic RV function, 9% had dilated left atrium, right atrium, RV, 14% tricuspid regurgitation, 4% aortic regurgitation, mitral regurgitation, 0% mitral stenosis, aortic root dilation - Final diagnosis of atrial fibrillation in 5%, sepsis 2.5%, 17.5% COPD, 15% Asthma, no abnormality found in 5%	- More feasible for rapid diagnosis, reduced time to diagnose in the emergency department - Improves diagnostic accuracy as an extension of physical examination
Emergency and Trauma				
Breitkreutz et al., 2010 [46]	Prospective	- Cardio-pulmonary resuscitation or in shock state	- 230 patients - 204 FEEL examination [Cardiac arrest (100) + shock state (104)] - 35% of those with an ECG diagnosis of asystole and 58% of those with pulseless electrical activity, coordinated cardiac motion was detected	Echocardiographic findings: - Altered management in 78% of cases - Associated with increased survival
Zanobetti et al., 2017 [33]	Prospective	- Evaluation of acute dyspnea	- 2683 patients - Average time needed to reach diagnosis was significantly lower - PoCUS was sensitive for diagnosing heart failure compared to COPD/asthma and pulmonary embolism	PoCUS is reliable for diagnosis of patients with dyspnea - Reduced diagnosis time
Nakao et al., 2020 [47]	Prospective	- Clinical impact on patients with acute heart failure and COPD	- 81 patients evaluated by lung PoCUS; 243 matched patients - Evaluated patients received treatment faster	- Faster administration of disease-specific treatment in elderly with suspected COPD or acute heart failure
Buhumaid et al., 2018 [48]	Prospective	- Diagnosing the cause of shortness of breath and chest pain - Comparative accuracy with a chest X-ray	- 128 patients with a mean age ± 17 years - Higher SP of PoCUS in all indications except pneumonia as compared to chest X-ray - Pneumothorax, pericardial effusion, and pleural effusion patients were correctly identified	- PoCUS is a highly effective test in the evaluation and diagnosis of chest pain and shortness of breath; narrows down the differential diagnosis - Chest X-ray has minimal clinical value in the setting of routine thoracic ultrasound
Becker et al., 2019 [39]	Prospective, multi-center	- Diagnosis of SBO	- 217 patients with an SBO prevalence of 42.9% - PoCUS for SBO showed SP 0.54 (95% CI = 0.45 to 0.63) and SN 0.88 (95% CI = 0.80 to 0.94) - Abnormal peristalsis and small bowel dilation of ≥ 25 mm were sensitive ultrasound parameters - Specific parameters are bowel wall edema, transition point, and intraperitoneal free fluid	- Moderate SN of PoCUS for SBO - Physician emergency ultrasound familiarity and training increase accuracy
Akyol et al., 2016 [38]	Prospective	- Diagnosis of shoulder dislocation	- PoCUS SP and SN in identifying dislocation were 84.2% and 100%, respectively - Confirmed reduction in 93 of 94 patients with 100% SP - 100% SN for excluding shoulder fracture, but 84.2% SP	- Effective tool to rule out or rule in shoulder dislocation in ED - High sensitivity for excluding fractures but with false-positive results
Weile et al., 2018 [49]	Prospective	- Ultrasound findings in unselected patients	- Positive findings in 39.3% of all patients - 62 positive examinations in 58 unique orthopedic complaint patients - 77 positive examinations among 59 unique patients with medical complaints - 55 positive examinations among 42 unique patients with surgical and abdominal complaints	- Positive PoCUS findings in >1/3rd unselected patients in ED
Tzadok et al., 2018 [50]	Observational	- Jugular vein ultrasound for acute dyspnea assessment	- Respiratory area changes of internal jugular vein had 70% accuracy in identifying acute decompensated heart failure in ED	- Ultrasound of internal jugular vein helps diagnose acute decompensated heart failure - Easy to measure and requires little skill; not affected by the habitus of the patient’s body.
Gungor et al., 2017 [51]	Prospective	- Acute appendicitis diagnosis	- 264 patients - 169 had a diagnosis of acute appendicitis - SN and SP were 92.3% for PoCUS	- PoCUS performed for diagnosis of acute appendicitis in ED has high SN and SP - Positive impact on clinical decision-making of ED physicians
Bothwell et al., 2014 [52]	Prospective	- Impact of decontamination therapy on ingested pills	- 37 ED physicians completed the study - All correctly identified the absence of tablets in bags containing only water - Diagnosed the presence of ECA tablets in bags containing water and PEG - For Part 2 of the study, most participants - 67.5% (25/37) using water, (62.1%) 23/37 using PEG, and all 100% (37) using activated charcoal - underestimated the number of ECA pills in solution by at least 50%	- Potential role of PoCUS in evaluating suspected acute, massive overdose in patients.
Graglia et al., 2021 [53]	Prospective	- To validate Bedside Sonographic Acute Cholecystitis (SAC) Score to diagnose acute cholecystitis	- 53 patients; the cutoff of ≥ 4 was used - Bedside SAC Score had an SN and SP of 88.9% and 67.5%, respectively - A Bedside SAC Score of < 2 had SN and SP of 100% and 35%, respectively - A Bedside SAC Score of ≥ 7 had SN and SP of 44.4% and 95.7%, respectively	- For diagnosis of acute cholecystitis, a bedside prediction score has great utility in ED - Rule out low <2 and high >7 scores
Prager et al., 2018 [54]	Prospective	- Remote mass gathering with limited resources	- PoCUS was used on 28 of 686 patients treated in medical tents to narrow the differential diagnosis in 64% of cases, alter working diagnosis in 21% of cases, a change management plan in 39% of cases - Reduce the burden on broader healthcare resource utilization in 46% of cases - Prevented ambulance transport off-site in 32% of cases - Absolute risk reduction of 1.3% in hospital-transferred patients	- PoCUS helped improve the diagnosis and management of patients at a remote music festival - Reduced ambulance transfers off-site and burden on healthcare
Gynecology and Obstetrics				
Ortner et al., 2019 [55]	Prospective	- To study the prevalence of cardiac dysfunction, PIS, and increased ONSD among patients with late-onset preeclampsia	- PIS, systolic dysfunction, LVEDP, and diastolic dysfunction were present in 24%, 10%, 25%, and 33% of women - ONSD was increased in 28% of women - No association between suspicious cardiotocography and PoCUS abnormalities - No association between albumin level, PIS, systolic dysfunctions, or raised LVEDP - PIS was associated with raised LVEDP and diastolic dysfunction - BNP levels were associated with diastolic and systolic dysfunction	- Diastolic dysfunction, increased ONSD, and PIS were common in preeclampsia with severe features - As compared to albumin level, cardiac ultrasound abnormalities are more useful for predicting PIS - Raised LVEDP was excluded by the absence of PIS - Cardiac ultrasound abnormalities had an association with BNP levels
Kodaira et al., 2021 [37]	Prospective	- Ultrasound findings using hand-held PoCUS	- Highly accurate for detecting free fluid collection in the abdominal cavity - Ultrasound findings are highly reliable for intrauterine pregnancy, cephalic presentation, fetal heartbeat, multifetal pregnancy, and gestational age assessment based on bi-parietal diameter - Least reliable for the detection of placenta previa or low-lying placenta	- Handheld PoCUS findings were found to be reliable in high-volume resource-limited hospitals for detecting urgent pre-specified obstetric findings
ICU				
Lu et al., 2020 [34]	Retrospective	- Management of high-risk patients in cardiac-surgical and cardiac-medical ICU by rescue PoCUS	- rescue PoCUS was performed on 141 patients - Common indications included hypotension, assessment of ECMO, arrhythmias, ventricular assist devices, abnormal pulmonary artery catheter values abnormality, and ischemic ECG - 129 examinations were positive for cardiac pathology - LV and RV dysfunction, hypovolemia, hypervolemia, pericardial effusion, and ECMO malposition were commonly diagnosed pathologies	- rescue PoCUS examination resulted in diverse diagnosis and management changes - 75% of examinations resulted in further interventions, such as fluid resuscitation (13%), diuresis (7%), inotropic support (12%), surgical intervention in the operating room (11%), bedside surgical intervention (4%), ECMO initiation (8%), and ECMO setting adjustment (6%)
Chen et al., 2018 [35]	Prospective	- Daily use in morning rounds; improvement in critically ill patients with sepsis	- 129 subjects at tertiary care hospital ICU; 88 in the control; 41 in the intervention group - In Univariate analysis intervention group had more negative fluid balance and shorter durations of mechanical ventilation on day three - In multivariable analysis, PoCUS was associated with a lower risk of prolonged ICU stay (>7 days)	- PoCUS during morning rounds was associated with a shortened duration of length of stay in the ICU and mechanical ventilation
Zieleśkiewicz et al., 2015 [56]	Prospective, multi-center	- Diagnostic and therapeutic use of PoCUS	- 1073 PoCUS/day on 709 patients in 145 ICUs - PoCUS served for procedural guidance in 13% of cases and diagnostic assessment in 87% of cases - Therapeutic and diagnostic impact of POCUS examinations were 69 and 84%, respectively	- PoCUS is highly useful for diagnostic assessment - Has a critical impact on the treatment of ICU patients
Pulmonology				
Shetty et al., 2021 [36]	Prospective	- To compare lung ultrasound patterns in third-trimester gravidas with and without preeclampsia	- 262 women with single pregnancies between 32-41 weeks of gestation - Among healthy gravidas, PoCUS was negative for pulmonary interstitial edema - Two patients with preeclampsia and respiratory symptoms had positive findings on PoCUS - One or two B-lines and three B-lines in one lung field in 18.6% of patients with preeclampsia and 11.4% of healthy gravidas were identified	- Lung ultrasound in women with preeclampsia without respiratory symptoms or signs of pulmonary edema are similar to patterns of healthy gravidas - PoCUS can be used to evaluate third-trimester gravidas with respiratory complaints, preeclampsia, or signs of pulmonary edema
Musculoskeletal, Skin, and Soft Tissues				
Crombach et al., 2020 [40]	Prospective	- Diagnostic value of PoCUS in the ankle; 5th metatarsal bone fracture	- 242 patients, with 35 having non-avulsion fractures, were diagnosed by radiograph - SN and SP in detecting fractures were 80% and 90.3%, respectively, by all sonographers;82.8% and 99.2%, respectively, by experts	- PoCUS, together with OAR (Ottawa ankle rules), has better diagnostic value as compared to radiography
Knaysi et al., 2020 [41]	Prospective	- Efficacy in detecting suspected skin and soft tissue infections	- 64 suspected patients were enrolled; 29 had PoCUS-proven abscesses, 33 had cellulitis, and 2 were excluded - Additional use of PoCUS had SN of 96.2%, SP of 93.9%, PPV of 92.6% - 10 of 62 patients’ management was changed; the most common change was a new incision and drainage or needle aspiration.	- PoCUS helped to rule in diagnosis and change management in patients with skin and soft tissue infections
ENT				
Gibbons et al., 2020 [42]	Retrospective	- Management of peritonsillar abscess	- Cohort 1 enrolled 48 patients; 12 had PoCUS; Cohort 2 enrolled 114 patients; 89 had PCoUS - EP successfully aspirated 89.1% with PoCUS vs. 24.5% without ultrasound - EP and ENT combined successful aspirations for PoCUS were 99.0% vs. 80.3% for ultrasound - ENT consultation with PoCUS use was 12.9% vs. 65.6% with ultrasound - CT usage with PoCUS was 23.8% vs. 37.7% with ultrasound - Return visits with PoCUS were 3.96% vs. 18.0% with ultrasound	- PoCUS use for peritonsillar abscess treatment improves aspiration, decreases CT consultations, LOS, and return visits
Pediatrics				
Doniger et al., 2018 [43]	Prospective	- PoCUS accuracy in diagnosing appendicitis	- 40 patients aged 2 to 18 years presenting with abdominal pain to pediatric ED - 16 (40%) had pathology-confirmed appendicitis - PoCUS had an SN and SP of 93.8% and 87.5%, respectively - Radiology-performed ultrasound had an SN and SP of 81.25% and 100%, respectively - Radiology-performed and PoCUS examinations had a very good agreement (κ = 0.83, p < 0.0005) - PoCUS identified all patients with an average score > 6 - Overall, the reduction in CT examinations was 55%	- In pediatric patients presenting with clinical concern for acute appendicitis, a staged algorithm that incorporates PoCUS is accurate and has the potential to decrease CT scan utilization
Freidman et al., 2019 [44]	Retrospective	- To determine agreement and time difference between PoCUS imaging by pediatric EP compared to radiology department imaging for children	- 75 patients; patients aged 0 to 18 years presenting to tertiary pediatric ED - In 58 of 75 cases, there was an agreement between PoCUS diagnosis and final diagnosis (κ = 0.71; 95% CI, 0.6-0.83) - In 25 of 28 cases, there was agreement in which pediatric EP performed PoCUS examinations with fellowship training in PoCUS (κ = 0.87; 95% CI, 0.72-1.00) - Results for PoCUS were generated in a median of 115 minutes (interquartile range, 68-185) before radiology department imaging results	- PoCUS imaging by pediatric EP for children with neck masses is a promising new POCUS application that may be able to save time in pediatric ED
Freidman et al., 2019 [57]	Chart review	To determine the accuracy of PoCUS by pediatric EP	- 120 patients; 12 cases of testicular torsion - For all causes of the acute scrotum, PoCUS agreed with the final diagnosis in 70% (95% CI 62-78%) of cases - More experienced PoCUS users displayed higher agreement with the final diagnosis - PoCUS results have generated a median of 73 minutes (interquartile range 51- 112) before radiology department ultrasound results	- PoCUS by pediatric EP is accurate for detecting testicular torsion in children with acute scrotum and could expedite diagnosis of this time-sensitive condition
Lin et al., 2018 [58]	Retrospective	- To determine if ED LOS differed for children who received PoCUS versus radiology-performed ultrasound	- Children presenting to urban pediatric ED between January 2011 and June 2013 with a diagnosis of cellulitis or abscess who underwent soft tissue ultrasound - Among 3094 children with a diagnosis of cellulitis or abscess, 202 underwent a PoCUS, and 118 underwent radiology-performed ultrasound - PoCUS group had a shorter median LOS than the radiology-performed ultrasound group (adjusted median difference 73 min; 95% interquartile range, 93.6 to 52.4) - In a subset of patients discharged from ED, this difference was more pronounced (adjusted median difference 89 min; interquartile range, 109.9 to 68.1)	- Among children presenting to a pediatric ED with superficial skin and soft tissue infections, children receiving PoCUS experienced shorter LOS compared to children receiving radiology-performed ultrasound

PoCUS use during COVID-19 pandemic

Multi-organ PoCUS, with other imaging and diagnostic modalities, played a significant role in triage, diagnosis, and management of COVID-19 patients. The prospective study by Alharthy et al. explains that PoCUS helped detect various pulmonary changes, such as B lines and pleural lines, in ICU-bound patients with deep venous thrombosis (DVT) [59]. The bedside lung ultrasound in emergency (BLUE)-protocol adopted from lung ultrasound is helpful for immediate diagnosis of acute respiratory failure and for reducing the radiation doses from ICUs to the point of care. BLUE protocol also has profiles designed with 90% accuracy for diagnosing primary diseases such as pneumonia, congestive heart failure, chronic obstructive pulmonary disease, asthma, pulmonary embolism, and pneumothorax [60]. In patients with COVID-19 pneumonia, lung ultrasound is a reliable alternative to thoracic CT scans [61].

According to a cohort study, the early detection of COVID-related pulmonary changes also helped to detect false positive reverse transcription polymerase chain reaction (RT-PCR) [62]. PoCUS aided in assessing and managing circulatory and hypoxic pulmonary failure in the ICU [63]. With an expanding scope during COVID-19, lung PoCUS emerged as an alternative and cost-effective diagnostic tool in step-ups where RT-PCR or CT chest is unavailable and can be an effective tool for DVT screening in COVID-19 patients, as explained by Brenner et al. and Galien et al. [64,65]. Bedside PoCUS, apart from aiding in cardiopulmonary and thromboembolic diagnosis, and detection of false positive RT-PCR, being a rapid and cost-effective alternative in resource scare settings, also played a role in triaging patients during the evolving times of the pandemic [59,62-64]. Not only is PoCUS recommended to monitor the fluid removal efficacy of diuresis or renal replacement therapy, but it even aids in predicting restrictive renal index and severity of acute kidney injury in severely ill COVID-19 patients [66,67]. The venous excess ultrasound (VExUS) evaluates IVC congestion and the severity of congestion in the liver, gut, and kidneys [5]. VExUS grading system predicts acute kidney injury by assessing the severity of venous congestion [68]. Therefore, a multi-organ PoCUS approach with other clinical and laboratory variables is also recommended in preference to X-ray and other imaging modalities in managing COVID-19 [66]. Table 4 explains the emerging role of PoCUS in the COVID-19 pandemic.

**Table 4:** PoCUS use during COVID-19 pandemic DVT: Deep vein thrombosis; ARDS: Acute respiratory distress syndrome; ED: Emergency department; RT-PCR: Reverse transcription polymerase chain reaction; LUS: Lung ultrasound; IVC: Inferior vena cava; LUZ: Lung ultrasound Zaragoza; NPV: Negative predictive value; PPV: Positive predictive value; AUC: Area under the curve; RRI: Renal restrictive index; AKI: Acute kidney injury; PoCUS: Point-of-care ultrasound

Study name, year	Study type	PoCUS use	Study details/outcome	Conclusion
Alharthy et al., 2020 [59]	Prospective	- Detection of pulmonary changes due to COVID-19; screening for DVT occurrence	- 89 confirmed COVID-19 cases in ICU of age >18 years; with serious pneumonia resulting in respiratory failure - B-Lines, pleural line irregularities, and variable consolidations were observed by PoCUS - DVT identification by PoCUS in < 20% of patients at the bedside, while CT-proven PE was found in 1/4th of patients	- PoCUS can be used as an alternative modality for ICU-bound COVID-19 patients to diagnose and monitor pulmonary changes and DVT detection
Bianchi et al., 2021 [62]	Prospective, single center	- Early detection; diagnostic stratification of COVID-19 related pulmonary changes; correspondence with positive RT-PCR; also, used to detect false negativity of RT-PCR	- ED patients with high-risk exposure to known COVID-19 cases in the past 14 days; at least one clinical criterion suspicious for COVID-19 infection; any radiological findings of pneumonia; minimum one LUS examination in ED - Patient with age < 16 years; pregnancy; known SARS-CoV-2 infection were excluded - Atypical B or C lines, multiple consolidations; ARDS with bilateral multifocal B pattern with shattered areas of A pattern were observed by PoCUS	- PoCUS is effective in diagnostic stratification during COVID-19 outbreaks - Helps in identifying false positive RT-PCR results, in turn improving diagnostic sensitivity in ED
Bitar et al., 2021 [63]	Prospective, single center	- LUS and echocardiography were performed within 12 hours of ICU admission	- Patients with age > 18 years; high suspicion of COVID-19 - LUS signs under evaluation were bilateral B lines, bilateral diffuse irregularities of pleural line, absence of significant pleural effusion, presence of multiple subpleural consolidations of various sizes - Echocardiography findings included E/A, deceleration time (DT), E/e left ventricular filling pressure, inferior vena cava collapsibility	- PoCUS plays an essential role in the assessment and management of acute hypoxic pulmonary failure and circulatory failure in COVID-19 patients
Brenner et al., 2021 [64]	Retrospective	- To compare 12 field LUS data with RT-PCR, which was designated as a gold standard investigation for diagnosis of COVID-19	- 174 patients from two urban tertiary healthcare centers - Criteria under consideration included pleural irregularity, multiple discrete B lines, confluent B-lines, subpleural consolidations, and pleural effusions	- Lung PoCUS is a rapid and cost-effective tool and can be used as an alternative diagnostic tool in resource-limited setups where RT-PCR or CT chest are unavailable
Garcia-Ceberino et al., 2021 [69]	Retrospective	- PoCUS diagnosed proximal and distal DVT; CT pulmonary angiogram was done to rule out pulmonary embolism	- 87 patients across the general ward and ICU - Depending upon risk factors for DVT, a lower extremity ultrasound was performed to screen for the occurrence of DVT	- PoCUS helps detect DVT in COVID-19 patients; however, it cannot rule out the presence of pulmonary embolism
Chen et al., 2020 [70]	Prospective	- Two ultrasound fellowship-trained ED physicians performed IVC, focused cardiac ultrasound (FOCUS), and LUS exams in patients with high clinical suspicion of COVID-19	- 96 patients at ED of two academic hospitals - PoCUS discovered irregular pleural lines in 63.2% of the study population, bilateral confluence in 17.5%, and isolated B-lines in 53.1%, which were associated with a positive RT-PCR test and correlated with interleukin-6 levels - IVC ultrasound negatively correlated with expiratory pO2 and inspiratory pO2 - Expiratory diameter assessed by PoCUS positively correlated with Troponin I	- PoCUS exam was effective and impactful in the diagnosis, management, and prognosis of patients with confirmed or suspected COVID-19
Falster et al., 2021 [71]	Prospective, single center	- Daily LUS examination to assess for consolidations and pleural effusions	- 83 patients admitted to COVID-19 unit - Daily LUS findings were correlated with initial chest X-ray and clinical deterioration	- LUS can be a valuable tool in continuously monitoring COVID-19 patients - Although an initial LUS at admission might not be able to predict ICU admission, ARDS, or death, a rapid increase in Mongodi LUS scores may be suggestive of lung parenchymal, pleural disease progression requiring supplementary diagnostics and treatment
Galien et al., 2021 [65]	Observational	- To screen for DVT with proximal compression ultrasound of lower extremities	- 90 COVID-19 patients in a mixed medical and surgical ICU	- Compared to a complete duplex ultrasound of lower extremities, PoCUS can be a resource-sparing alternative for screening of DVT in COVID-19 patients
Gibbons et al., 2021 [72]	Prospective, single center	- Comparing radiological effectiveness of LUS with chest X-ray for diagnosing COVID-19 pneumonia	- 110 patients in an urban, academic, Level I ED with predefined signs and symptoms suggestive of COVID-19 for LUS	- LUS was found to be more radiologically sensitive than chest x-ray to diagnose viral/atypical pneumonia - Although LUS and chest X-ray both have low specificity, PoCUS can become a practical first-line imaging
Gonzalez et al., 2021 [73]	Observational	- Comparing right and left ventricular global longitudinal strain (GLS) with classic echo-doppler systolic function indices	- 30 COVID-19 patients in ICU - Echocardiography was performed on the day of admission, then on day three and day seven - Patients admitted for acute myocardial infarction or pulmonary embolism with a positive COVID-19 PCR swab test were excluded from study	- Ejection fraction for left ventricle (LVEF) and Left ventricular GLS are equivocally effective in assessing left ventricular systolic function - Right ventricular GLS weakly correlated with fractional area change (FAC), tricuspid annular plane systolic excursion (TAPSE) and not correlated in any way with tissue Doppler velocity of the basal free lateral wall (S’) for the right ventricle
Gracia et al., 2021 [74]	Prospective	- To analyze the prognosis of COVID-19 patients by LUZ-scale by LUS during the initial 72 hours of hospital admission	- 130 COVID-19 inpatients; a baseline LUZ score was established with primary endpoints being in-hospital death and/ or admission to ICU - Secondary outcomes like the length of hospital stay, escalation of medical treatment, and oxygen requirements were predicted using the baseline score	- Baseline LUZ score was predictive of severity of infection during hospital stay and, in turn, helps in early intervention and prevention of adverse outcomes
Gutsche et al., 2021 [75]	Prospective	- To assess PPV, NPV of LUS compared to RT-PCR and clinical findings	- 101 suspected COVID-19 inpatients of > 18 years old; non-pregnant patients with specific COVID-19 symptoms - LUS was performed within 48 hours of admission and compared with a prospective RT-PCR test - LUS scoring ranged from 0-3 in 12 lung regions bilaterally based on ultrasound findings - Patients with a score of 0 or 1 in all areas were classified as "LUS-COVID-negative." - Patients with a score of 2 or 3 in 1 out of 12 areas were classified as "LUS-COVID-positive."	- PPV and NPV of LUS were assessed to be 34.1% and 97.1% - Significantly higher NPV of LUS can depict its importance in ruling out COVID-19 infection
Kapoor et al., 2021 [76]	Prospective	- To screen for and diagnose lower extremity deep venous thrombosis on Days 1, 7, and 14	- 107 ICU COVID-19-positive patients confirmed by RT-PCR - The "2-region technique" was implemented for the diagnosis of DVT in the lower extremity, which involved compression of 1-2 cm area proximally and distally to (i) junction of great saphenous vein and common femoral vein extending to the confluence of superficial and deep femoral vein, (ii) posterior aspect of knee extending from proximal popliteal vein to confluence of calf vein	- PoCUS diagnosed 15.9% lower extremity DVT on Day 1, 6% on Day 7, and 4.1% on Day 14 - PoCUS is helpful in screening and diagnosing thromboembolic events during a pandemic, even in a setting with limited resources
Lieveld et al., 2021 [77]	Prospective	- Correlation of LUS findings; severity staging compared to CT severity score	- 114 RT-PCR confirmed COVID-19 patients - six lung fields were examined bilaterally in all patients in the ER, and a cutoff score of 12 was decided - Time to poor outcome was shorter in study group with LUS > 12 compared to that of LUS < 12	- Discriminative ability of one ultrasound was similar to that of the CT severity score in determining hospital admission (ward or ICU) or no admission with a statistically significant AUC of 0.83 (95% CI, 0.75-0.91)
Narinx et al., 2020 [78]	Retrospective	- To evaluate the accuracy, NPV, and PPV of lung PoCUS as compared to RT-PCR and CT	- 93 patients - Three BLUE points in each hemithorax were examined in either supine or semi-recumbent position for B-line pattern and thickening of the pleural line	- SN (93.3%) and NPV (94.1%) were significant compared to RT-PCR. But, SP (21.3%), PPV (19.2%), and accuracy (33.3%) were lower compared to RT-PCR - Significant values for SN (80.6%), SP (86.7%), NPV (95.6%), and accuracy (85.6%) were obtained, and a moderate value for PPV (54.5%) when compared to CT scan
Renberg et al., 2021 [67]	Observational	- To describe the pattern of RRI concerning AKI in COVID-19 patients	- 51 COVID-19 patients in ICU - Patients were examined in the ICU in supine or prone positions based on position favorable for maximum ventilation - Kidneys were examined bilaterally, and RRI was assessed on both sides	- Bedside PoCUS examination can measure RRI and, in turn, predict the severity of AKI in severely ill COVID-19 patients

PoCUS in outpatient and innovation

Table 5 describes the use of PoCUS in outpatient settings.

**Table 5:** PoCUS in the outpatient setting ED: Emergency department; OP: Outpatient; LV: Left ventricle; RV: Right ventricle; LAE: Left atrial enlargement; IVC: Inferior vena cava; SN: Sensitivity; SP: Specificity; MACE: Major adverse cardiovascular events; PE: Pulmonary embolism; AAA: Abdominal aortic aneurysm; FAST: Focused assessment with sonography in trauma; PoCUS: Point-of-care ultrasound

Study name, year	Study type	PoCUS use	Study details/outcome	Conclusion
Kovell et al., 2018 [79]	Literature review	- Outpatient: risk stratify and diagnose medical condition- cardiovascular disease - ED, critical care: guide triage and critical care interventions	- Early identification and help direct management decisions - OP setting: 84% 2D echocardiogram pick up 82% abnormal cardiac finding compared to 47% by physical exam - ED: PoCUS had a higher ICU admission rate (80.4 vs. 67.2%), decreased mortality (11% vs. 19.5%), an essential tool to guide the management of shock - ICU: 28-day survival improved 66% vs. 56% standard of care	- PoCUS can be a useful adjunct to physical exams, particularly in critical care applications
Kobal et al., 2016 [80]	Prospective, multi-center	- Triage of cardiac disease by eyeball examination of global and regional wall motion abnormality - Color Doppler to diagnose valve malfunction - Severity of valve regurgitation by jet area - Liner measurements of LV, RV size, LV wall thickness, ascending aorta	- Of 207 OP and inpatients, 56% were males - 71% in ICU, 17% outpatient, 12% floors - After PoCUS, 14% of physicians changed their diagnosis, 9% from cardiac to non-cardiac; 3% changed etiology of cardiac disease, 2% changed from non-cardiac to cardiac - In 52% change of diagnosis or treatment plan, 39% changed assessment to perform another diagnostic study, 5% referred to the therapeutic procedure, 2% decided against the procedure, 8% were hospitalized, 1% discharged - PoCUS helped 48% of patients in triage, 33% with minor contribution, 19% with no impact - 14% change in primary diagnosis, 48% upgrade in diagnosis after PoCUS	- Extension of physical examination with PoCUS by cardiology fellows and cardiologists in the triage of patients with acute cardiac problems resulted in alteration of diagnosis, change in management in >50% of patients, improved the confidence level of physician - PoCUS can prevent unnecessary additional diagnostic studies, guide patient therapy, hospitalization and discharge
Han et al., 2019 [81]	Retrospective	- To refine referral for OP Echocardiography	The referral model has been developed after identifying LAE, age, and diabetes in patients who ultimately showed significant echocardiogram abnormalities and higher long-term mortality - Initial derivation cohort on 233 pts with mean age 63+/- 17 yrs., 45% male, with LAE had 70% accuracy, 58% SN, 78% SP and IVC plethora had 71% accuracy with 34% SN, 94% SP - In the derivation cohort, MACE or death at 5.5 years was 38% abnormal vs. 13% in the normal echocardiogram - Prevalence of LAE, IVC plethora sign and both were 43%,10%, and 7%, respectively - Mortality at 5.5 years was 14.6% overall, and in pts with LAE 23%, IVC plethora sign 25% and 38%. In those lacking both signs, overall mortality was 8%	- Echocardiogram ordered by OP physicians often turns normal - Referral for Echocardiogram after initial screening PoCUS provides prognostic information, is a cost-effective care
Ahmad et al., 2022 [82]	Case Report	- To rapidly diagnose massive PE and DVT in daycare or OP setting	- Left femoral vein thrombus; increased right heart pressure in PoCUS led to the decision to administer fibrinolytic therapy - PoCUS allowed for rapid diagnosis of PE; institution of anticoagulant therapy to save life - PoCUS in lower limb showed absence of doppler signal, lung showed A-profile bilateral means unremarkable, cardiac PoCUS revealed dilated RV, flattening of septal wall, D shaped LV - Lower limb PoCUS helped in increased predictive performance in diagnosing PE	- Lung, lower limb, and cardiac PoCUS helps in identifying PE, especially in hemodynamically unstable patients - PoCUS is a handy tool useful in day-care surgery or OP setting
Frasure et al., 2020 [83]	Literature Review	- Facilitates triage patients; provides diagnostic information quickly in OP, urgent care, or ED - Use for soft tissue infections, gallstones, AAA, kidney stones, intrauterine pregnancy; diagnosis of foreign bodies	- High-frequency transducers (linear probe) used for superficial structures up to 6 cm depth like skin, muscle and bones - Lower frequency probes (phased array, curvilinear transducers) penetrate deeper up to 20-30 cm, used for less detailed and deep structures like AAA - Diagnosis of abscess, especially within cellulitis, cholelithiasis, not only kidney stone but also hydronephrosis, quantification of size of AAA in addition guiding removal of foreign body and identification of intrauterine pregnancy even as early <4 weeks or ectopic pregnancy is possible with PoCUS use - In traumatic conditions, pneumothorax, hemothorax, pericardial effusion, retinal detachment, lens dislocation, and retinal/ vitreous hemorrhage can also be diagnosed with PoCUS - PoCUS reduces the length of stay from 26 minutes to 2 hours; also affordable for urban or rural smaller size facilities like primary care, family medicine or intensive care, internal medicine - Also helpful in performing peripheral intravenous lines, paracentesis, thoracentesis, and lumbar punctures.	- Integration of PoCUS into clinic/outpatient settings is helpful as a bedside tool for diagnosis and expedition of medical treatment.
Hamill et al., 2020 [84]	Prospective	- To diagnose thyroid nodules and goiters by trained non-radiologist	- Total 40 scans between January 2017 and April 2018 with a mean age of 52 years, 30 females, 10 males; indication nodules in 20, goiter in 13, patient preference in 7; British thyroid association classification U scoring U1+U2 in 37 patients, U3-U5 in three patients - Duration of scan 5-7 minutes - All reporting of POCUS performed by endocrinologists reviewed by radiologists - Fine needle aspiration biopsy result Thy1 in 2, Thy2 in 6; Thy 5 in one patient and diagnosis benign in 39 and malignant in one patient	- PoCUS can be useful in terms of same-day scanning, non-invasiveness, cost, and time-saving, expediting diagnosis, easing the burden on radiologists, and enhancing patient satisfaction in endocrine OP settings
Arya et al., 2022 [85]	Retrospective, single-center	- Diagnosis made with POCUS in specialist palliative care setting	- PoCUS was used for 126 conditions in 89 patients - 61 (48.4%) in OP settings; 75% had been diagnosed with cancer - 81 (64.3%) ascites, 21 (16.7%) pleural effusion, 2 (1.5%) bowel obstruction, 1 (0.8%) pneumothorax, and 2 (1.5%) congestive heart failure using PoCUS - 52 paracentesis and 7 thoracentesis performed using PoCUS - 57 (45%) use of PoCUS in the palliative care unit, 30 (24%) in the clinic, 22 (17%) at home, 7 (6%) long-term cancer center	- PoCUS aids in diagnosis management in inpatient and outpatient settings; reduces visits to hospital; decreases the number of hospital deaths; reduces complications of procedures; better symptom control; improves quality of life and patient satisfaction
Rominger et al., 2018 [86]	Prospective, multi-center	- Longitudinal POCUS training provided to physicians in four different sessions over one year for rural physicians in Mexico	- Over 12 months period, 584 ultrasound, 45.5% transabdominal obstetric, 26.6% abdomen/pelvis, 5.7% musculoskeletal, and 5.7% skin and soft tissue examination performed - Use of PoCUS changed the diagnosis in 194 (34%) patients and management changed in 171 (30%) patients	- PoCUS training is a useful tool for rural physician to improve their clinical outcome
Sullivan et al., 2021 [87]	Retrospective, single-center	- As a diagnostic modality in the military for a month-long Brazilian Riverine mission in February 2019	- PoCUS was used in 24 (3%), 88% of females - Of females, 21 (52%) were pregnant; 14 (54%) were pelvic obstetric cases; 3 (12.5%) non obstetric pelvic; 4 (16%) FAST/abdominal examination - Out of 24 who underwent PoCUS examination, 10 (42%) had symptoms; without PoCUS, those patients might have been recommended for evacuation (referral to a higher center) - In each case, PoCUS provided viable intrauterine pregnancy as an explanation of amenorrhea or showed absence of concerning pathology that prevented OP primary care visits	- PoCUS proved an inexpensive, effective tool for preventing unnecessary referrals. It is a benefit advantage of adding PoCUS to humanitarian assistance missions

PoCUS can be used for heart failure outpatient clinic volume status assessment [88]. PoCUS has 73% sensitivity and 75% specificity in diagnosing left ventricular hypertrophy (LVH), thus helping to screen LVH patients or modifying antihypertensive therapeutics [89]. The REASON trial on patients with pulseless electrical activity showed that PoCUS was associated with higher survival rates to admission and discharge [90]. In patients with chest pain after initial EKG, PoCUS is used as a helpful adjunct to diagnose pericardial effusion, regional wall motion abnormalities, and type A aortic dissection [91]. However, PoCUS has limitations in diagnosing pulmonary embolism [92]. Compared to a 2D echocardiogram, PoCUS has 98% specificity but only 26% sensitivity in diagnosing right ventricular strain [93]. Additionally, with the availability of new technology, images can now be shared with colleagues/specialists without time-consuming uploads to EMR [94].

Benefits and limitations of PoCUS 

PoCUS is an innovative, portable, and cost-effective solution to imaging that plays a vital role in reducing the number of clinicians and the constant need for patient transfer in in-patient settings. Thereby creating a streamlined environment [95]. An improved doctor-patient relationship reported by 45% of patients increased patient trust in the physician's assessment and consultation. It helped patients become more aware of their health condition, as 85% of patients reported in a cross-sectional study that patients felt more secure, and 95% reported improved service and quality of care [96]. Having played a major role in clinical practice, PoCUS demonstrates improved diagnosis and sensitivity compared to ultrasound and computed tomography for various cardiopulmonary, gastrointestinal, obstetrics, gynecological, and musculoskeletal conditions, with great specificity in COVID-19 patients. It helped integrate sonography with clinical examination and history at the bedside [62,97].

Due to the ease of use and high sensitivity, PoCUS is formally being introduced in the medical education curriculum at various programs. Helping in developing the skills of students in clerkships [97]. Despite having numerous benefits, the need for standardized training availability for PoCUS and lack of enrollment, the desire to achieve competency impacts the proficiency and efficiency of healthcare workers, the knowledge gap in image interpretation, and the need for more supervision limits this technology's benefits. With only a fraction of doctors needing more confidence about their skills, according to a survey, and increasing usage of PoCUS in areas not usually recommended, such as aortic valve disease, the limitations of PoCUS are evident [98]. Table 6 describes the benefits and limits of PoCUS.

**Table 6:** List of benefits and limitations of PoCUS PoCUS: Point-of-care ultrasound

Benefits	Limitations
General: Portable, cost-effective, quick results	Knowledge gap
Clinical: Cardiopulmonary system, gastrointestinal system, obstetrics and gynecology, musculoskeletal, pediatrics, COVID-19	Lack of training; practice to maintain competency
Medical education	Lack of supervision
Improved patient-doctor relationship	Lack of standardized approach and coursework

Future directions 

The skills required for PoCUS usage and interpretation, resulting in limitations, will call for a novel standardized curriculum, supervision, and skill improvement that facilitates continuous hands-on training [99]. On the other hand, the advancement of medicine and the ever-expanding use of PoCUS in various specialties will require further advancement in usage and technology to meet the demands of changing times. With the integration of AI, cloud-based databases, 5G-based tele-remote usage, and robotics, the specialized PoCUS will facilitate the field of medicine with its advanced application [100].

Tele-remote ultrasound - using modern computer and network communication to digitalize the ultrasound images to achieve image transmission and storage remotely. It allows remote interventional procedures and diagnosis through audio, video, and text synchronization. This remote system enables doctors to use their skills to control the robot remotely to perform ultrasound scans and medical diagnoses based on the scanning [101]. 5G technology has met the long-distance, high-bandwidth, and high-resolution requirements for remote ultrasound consultation and operation, even proving great value during the COVID-19 pandemic for evaluating lung lesions and guiding procedures. Therefore, it is a promising direction for PoCUS [101].

The expanding field of AI technology is gradually being implemented in medicine. It alleviates human burden and allows fast imaging processing, handling massive data, efficient diagnostic imaging, and standard maintenance, which are efficient ways of optimizing PoCUS US image processing, analysis, and diagnosis [102]. With the convolutional neural network used by Buda et al. to develop an intelligent recommendation algorithm for the biopsy requirement of thyroid lesions, AI can play a vast role in ultrasound and PoCUS imaging [103]. The cloud-based data sharing of the US system, where the collected image data through 5G technology is transmitted between the two locations and utilized for consultation and diagnosis, is an option that we can explore further together with specialty-specific PoCUS for clinicians to have a better understanding of different organ system and their hemodynamic [104,105].

## Conclusions

PoCUS has emerged as a great tool, a cost-effective, portable, and efficient alternative to traditional ultrasound and CT scans. Its expanding usage beyond the emergency room into various specialties and settings played a significant role in the COVID-19 pandemic. By assisting in remote and onsite COVID-19 diagnosis, PoCUS has gained considerable space in medical school and training program curriculums. Despite having a few limitations due to a lack of standardized training, the emerging technological advancements in AI, 5G communication, and cloud-based sharing are promising futuristic directions for PoCUS imaging and medical diagnosis.
